# Preparation and Characterization of a Novel Aspirin Derivative with Anti-Thrombotic and Gastric Mucosal Protection Properties

**DOI:** 10.1371/journal.pone.0098513

**Published:** 2014-06-03

**Authors:** Xi-E Zhen, Ming Zong, Sai-Nan Gao, Yong-Gang Cao, Lei Jiang, Shu-Xin Chen, Kuan Wang, Shi-Qin Sun, Hai-Sheng Peng, Yu-Hua Bai, Sen Li

**Affiliations:** Department of Pharmaceutics, Daqing Branch, Harbin Medical University, Daqing, China; Univ of Bradford, United Kingdom

## Abstract

The use of acetylsalicylic acid (ASP) is limited by its adverse effects, especially the effect on the gastric mucosa. To address this problem, we synthesized a derivative form of ASP, prepared by modification of ASP with nano-hydroxyapatite (a kind of inorganic particle containing Ca^2+^). The derivative was named Ca-ASP. Structural study showed that Ca-ASP was a kind of carboxylate containing intramolecular hydrogen bonds. Rats given a high dose of Ca-ASP (5 mmol per kg body weight) showed similar anti-thrombotic activity as those given the same dose of ASP, but had much lower gastric mucosal damage than ASP (UI: 2 versus UI: 12.5). These rats also showed reduced expression of COX-2, but their COX-1 expression was similar to that of control rats, but significantly higher than that of ASP-administered rats. Furthermore, the level of prostaglandin E2 (PGE2) was up-regulated in Ca-ASP-administered rats compared to ASP-administered rats. Taken together, the results showed that Ca-ASP possessed similar antithrombotic activity as ASP but without the side effect associated with ASP, and the underlying mechanism may center on inhibiting COX-2 without inhibiting COX-1, and thus favouring the production of PGE2, the prostaglandin that plays a vital role in the suppression of platelet aggregation and thrombosis, as well as in the repair of gastric damage.

## Introduction

Acetylsalicylic acid (ASP) is a nonsteroidal anti-inflammatory drug (NSAID) that has long been used as an anti-pyretic and analgesic agent. It is also used to prevent and treat thromboembolic diseases [1]–[2]. Research has shown that ASP can be used to treat and prevent cancers, such as breast cancer [3], colon cancer [4] and rectal carcinoma [5]. So the use of ASP has become widened. However, despite the indisputable success of this drug, its use is associated with serious gastrointestinal mucosal damage [6]. A number of factors are believed to be responsible for the side effects of ASP. Firstly, ASP inhibits cyclooxygenase-1 (COX-1), an enzyme that is largely expressed in gastric epithelial cells and is involved in the synthesis of prostaglandins [7]. Prostaglandins play an important role in protecting the gastric mucosa [8]. Secondly, the free carboxylic group in ASP exerts a local irritant effect on the gastric mucosa. To overcome these side effects, ASP derivatives have been investigated as a possible replacement for ASP, and some ASP derivatives appear to offer promising results, such as ASP-NO [9] and ASP-H_2_S [10]. These two types of derivative release NO or H_2_S gas *in vivo*, and these gases help to protect the gastric mucosa. However, it is difficult to control the concentration of NO and H_2_S, and once either of the gas exceeds the normal range, it becomes toxic to some viscus [11]. Metal ions modified ASP (Metal-ASP) has also been developed on the basis that trace metal elements are essential nutrients for the human body. Metal-ASP such as V-ASP [12] and Cu-ASP [13] have been reported. However, the function and mechanism associated with the protection of gastric mucosa provided by Metal-ASP is still unclear.

Hydroxyapatite (Hap) is a component of the bones and has good biocompatibility and low cytotoxicity, and it has been used as delivery vehicles for different drugs [14] and genes [15]. Some researchers have demonstrated that Hap can up-regulate the level of prostaglandin 2 (PGE2) in the tissue [16]. Based on these findings, we modified ASP with Hap to obtain a derivative that could have less or no side effect on the gastric mucosa while retaining the antithrombotic property of ASP. The mechanism associated with gastric mucosal protection afforded by the Hap-modified ASP derivative was also investigated.

## Materials and Methods

### Drugs and chemicals

ASP was obtained from Aladdin Chemistry Co. Ltd, and Hap (20 nm) was bought from Nanjing Emperor Nano Material. All chemicals used in the study were of analytical grade.

### Synthesis and structural analysis of Ca-ASP

ASP (3.0 g) was added to 1 L of distilled water and the mixture was initially homogenized in an ultrasonic bath for 30 min at 15°C. The mixture was then placed on a magnetic stirrer and stirred until the ASP was completely dissolved. Hap (6.0 g) was added to 60 mL of distilled water and the mixture was stirred on a magnetic stirrer. The ASP solution was slowly added to the Hap suspension (while stirring) until the mixture changed from turbid to pellucid. A few more drops of ASP were added to the sample to ensure that ASP was in excess. The resulting solution was concentrated using a rotary evaporator and allowed to crystallize at 4°C. Since free ASP would also crystallize under this condition, but could be dissolved in ethyl acetate, the resulting crystals were dissolved in ethyl acetate to remove the free ASP crystals. The resulting insoluble crystals, which were the target product, were collected. The composition and structure of the compound were characterized by X-ray diffraction (XRD), scanning electron microscopy (SEM), and Fourier transform infrared spectrometry (FTIR). XRD was performed with a Rigaku D-MAX 2500PC X-ray powder diffractometer on finely powdered samples using Cu *Kα* radiation (40 Kv and 30 mA). Data were collected for 2*θ* values ranging from 5° to 40° at a scanning speed of 0.02° sec^−1^. SEM was performed with a scanning electron microscope (Jeol JEM 6400) equipped with an INCA-300 energy dispersive spectroscopy (EDS) system. The sample was glued onto a metal sample unit and then coated with gold. FTIR was performed with an infrared spectrometer (Perkin Elemer Spectrum One B) equipped with a heating cell with temperature control. The spectrum of the sample was measured with a KBr disk. Finally, the molecular mass of Ca-ASP was determined by LC-MS using a Waters platform ZMI LC-MS instrument. LC was carried out with a lichroshpher C-18 column (2.1×250 mm) using the following conditions: mobile phase from 10% methanol-90% ammonium acetate solution (0.02 M), column temperature of 30°C, and flow rate of 0.3 mL/min. MS was performed using positive ion electrospray ionization (ESI^+^) scan mode that was set to the following conditions: 120°C for ion source temperature, 300°C for desolventizer temperature, 30 V for capillary voltage. The sample was dissolved in DMSO and filtered through 0.45 µm filter prior to analysis.

### Animals experimental

Wistar rats of either sex (180–200 g in weight) were obtained from the Central Animal Facility of Harbin Medical University. The rats were housed in cages at ambient temperature and provided with food and water ad libitum. The animals were acclimatized to the laboratory conditions and fasted for 24 h before the experiment. Thirty rats were randomly divided into five groups (six animals per group). The negative control group was administered 0.5% Carboxyl Methylated Cellulose (CMC) aqueous solutions whereas the ASP group was administered ASP (dissolved in 0.5% CMC aqueous solution) at a dose of 0.5 mmol/kg of body weight. The remaining three groups were each given a different dosage of Hap-modified ASP (0.1, 0.25 or 0.5 mmol/kg, also dissolved in 0.5% CMC aqueous before gavage). After treatment for 20 d, blood samples were withdrawn from the retro-orbital cavernous sinus of the animals for the analysis of antithrombotic activity. The rats were euthanized with an overdose of chloral hydrate, and their stomachs were immediately excised and opened along the greater curvature, and the mucosal surface was observed under the anatomical lens. After observation, the stomachs were kept at −80°C for the analysis of COX expression and prostaglandin E2 (PGE2) content. All animal experiments were conducted in accordance with international ethical guidelines and the experimental protocols for using rats have been reviewed and approved by the Animal Ethics Committee at Harbin Medical University.

### Experiment of platelet count

Peripheral blood (20 µL) was added to 380 µL of ammonium oxalate diluents and the mixture was allowed to stand at room temperature for 10 min. Aliquot (10 µL) of this mixture was then placed in a cell counting chamber and allowed to stand at room temperature for a further 15 min. The platelets from five small squares within a large central square of the counting chamber were counted under the microscope. Platelet count was expressed as number of platelet per µL blood.

### Gastric damage score

Gastric mucosa ulcer index (UI) was determined according to the Guth standard [17]. The length and the width of the injured gastric mucosal tissue were measured with a vernier caliper. Spot erosion was recorded as 1 point, erosion length <1 mm was recorded as 2 points, between 1–2 mm was recorded as 3 points, and between 2–3 mm was recorded as 4 points, and >3 mm was recorded as 5 points. The score was doubled if the erosion width was >1 mm.

### Western blot analysis of COX-1 and COX-2

COX-1, COX-2 and β-actin (internal standard) proteins in the gastric mucosa were analyzed by western blot. A sample of the stomach tissue was homogenized in RIPA buffer in an ice bath. Protein concentration was measured with the BCA method and 50 µg of total protein was resolved in 10% SDS-polyacrylamide gel under reducing condition. After electrophoresis the proteins were transferred onto nitrocellulose membrane. The membrane was blocked with TBS-T containing 5% non-fat milk for 3 h and then incubated with a 1∶200 dilution of antibody against COX-1 or COX-2 or a 1∶5000 dilution of antibody against β-actin for overnight at 4°C. All antibodies were obtained from Boster Biological Technology, LTD, Wuhan. After four successive washes, the membrane was incubated with horseradish peroxidase-conjugated goat anti-rabbit (for anti-COX-probed membrane) or goat anti-mouse immunoglobin (anti-β-actin-probed membrane) at a dilution of 1∶10000. The blot was detected with chemiluminescence (Haigene Biological Technology., LTD., Harbin). The bands representing COX protein and β-actin were quantified by densitometry using a scanning software.

### Measurement of prostaglandin E2

Each sample of the gastric mucosa was weighted, minced with scissors, and homogenized in 0.9% sodium chloride at 4°C. The homogenates were centrifuged at 845×*g* for 10 min. The supernatants were subjected to PGE2 assay using a PGE2 Enzyme Immunoassay Kit (Boster Biological Technology., LTD., Wuhan). The concentration of PGE2 in the sample was expressed as ρg/mL of gastric mucosal tissue.

### Statistical analysis

Data were analyzed by Student *t* test or one-way analysis of variance (ANOVA) followed by Dunnet's multiple comparisons post hoc test. Data were expressed as means ± SDs.

## Results

### Structure and composition of Ca-ASP

**Figure 1:**
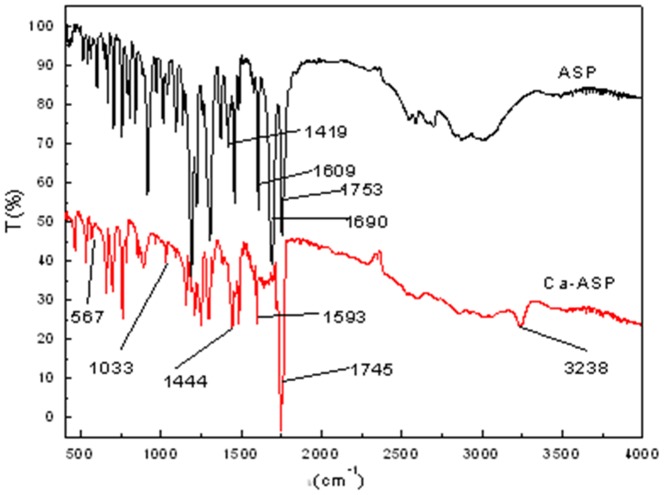
FTIR spectra of ASP and Ca-ASP.

The corresponding FTIR spectrum of Aspirin and Hap-modified ASP (henceforth referred to as Ca-ASP) are shown in Figure 1 and their characteristic FTIR vibrations are listed in Table 1. For Aspirin, the main characteristic absorption peaks were clearly present, such as those at 1753(s) cm^−1^, 1690 cm^−1^ and 2500–3200 cm^−1^, which represent, respectively, acetyl group [C = O(CH_3_)], carboxylic acid group [νC = O(-COOH)] and hydroxyl group [18]. For Ca-ASP, anti-symmetric stretching vibration peak of ν_as_COO^-^ shifted to lower wave number (from 1609 cm^−1^ to 1593 cm^−1^), while the symmetric stretching vibration peak of ν_s_COO^-^ shifted to higher wave number (from 1419 cm^−1^ to 1444 cm^−1^), and the peak at 1690 cm^−1^ [νC = O(-COOH)] disappeared compared to the FTIR spectrum of ASP. This indicated the formation of a carboxylate [19]–[20]. On the other hand, the absorption peak of the carbonyl (acetyl) group [C = O(CH_3_)] in Ca-ASP also shifted to lower wave number (1753 to 1745 cm^−1^), and the absorption peak of hydroxyl (νOH) shifted to 3238 cm^−1^, indicating that a hydrogen bond was formed between the acetyl and hydroxyl groups [21]. Additional FTIR spectra were measured at 100°C and 200°C (data not shown), which indicated the strength and position of peak representing the acetyl group [C = O(CH_3_)] and hydroxyl (νOH) groups were not changed, and therefore the hydrogen bond between the acetyl group and the hydroxyl group was an intramolecular bond [22]. There were two new absorption peaks, one at 1033 cm^−1^ and the other at 567 cm^−1^, which were due to absorption by phosphate group. The analysis showed that Ca-ASP was derived from Hap, which formed a kind of carboxylate via reaction with ASP, resulting in the formation of an intramolecular hydrogen bond.

**Table 1:** Characteristic FTIR vibrations for ASP and Ca-ASP.

ASP (cm^−1^)	Ca-ASP(cm^−1^)	Assignments
2500–3200	3238	associating υOH
1753	1745	νC = O(-C(O)OCH_3_)
1690	—	νC = O(-COOH)
1609	1593	ν_as_COO^-^
1482	1482	PhH, νCC, H_3_CCO, CH_3_
1458	1458	H_3_CCO, CH_3_
1419	1444	ν_S_COO^-^
1305	—	νC = O(-COOH)
—	1033	PO_4_^3-^
—	567	PO_4_^3-^

**Figure 2:**
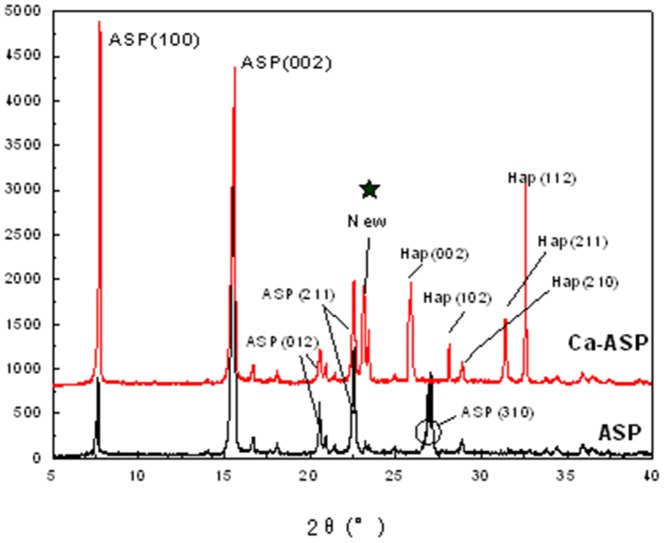
XRD spectra of ASP and Ca-ASP.

The spectra of ASP and Ca-ASP obtained from XRD analysis are shown in Figure 2. In the case of ASP spectrum, five main peaks with 2*θ* values of 7.7°, 15.6°,20.6°, 22.6° and 27.07°, corresponding to the characteristic crystal planes of ASP (100, 002, 012, 211, 310) [23], and the first four of these peaks were also present in the spectrum of Ca-ASP. However, the peak of 27.07° was absent in the spectrum of Ca-ASP and noticeable change in intensity occurred at 7.7° and 15.6°, which became stronger and weaker, respectively, compared to the spectrum of ASP. The spectrum of Ca-ASP also yielded five different peaks: 2*θ* values of 25.9°, 28.1°, 28.9°, 31.5°, and 32.9° corresponding to the (002), (102), (210), (211) and (112) crystal planes of hydroxyapatite [24]. Additional peak was also present at 22.6° in the spectrum of Ca-ASP. The XDR spectrum of Ca-ASP indicated that Ca-ASP was not simply a mixture of ASP and Hap, but was in fact a distinct crystal structure formed by reaction of ASP with Hap [25].

**Figure 3:**
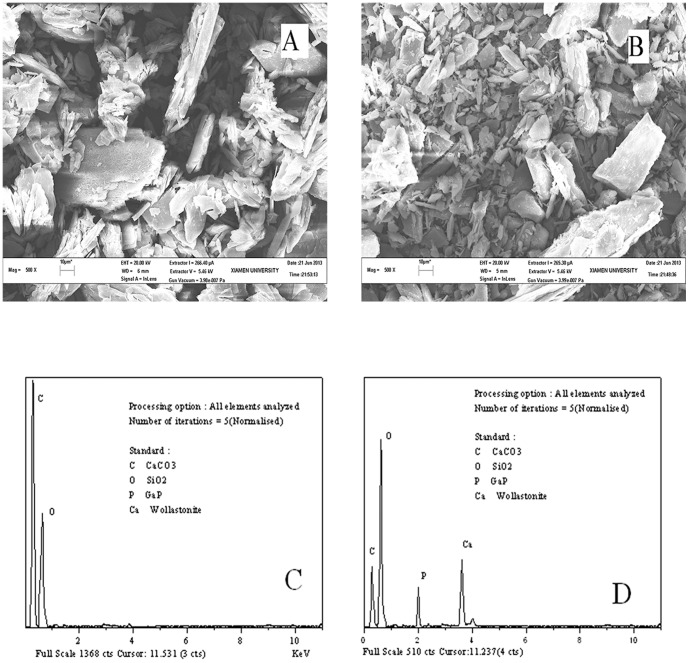
SEM micrograph of (A) ASP, (B) Ca-ASP and EDS spectrum of (C) ASP, (D) Ca-ASP.

The SEM micrographs of ASP and Ca-ASP are shown in Figure 3A and 3B, respectively. Both ASP and Ca-ASP had very similar crystalline structures as seen from the SEM micrographs of the two compounds. Elemental analysis of ASP and Ca-ASP was performed by EDS spectroscopy and the EDS spectra are shown in Figure 3C and 3D. The percentage of each element in the compounds is listed in Table 2. A carbon to oxygen ratio of 2.23∶1 was obtained for ASP, which was almost the same as the theoretical value of 2.25. Ca-ASP on the other hand showed a carbon to oxygen ratio of 0.323∶1 and calcium to phosphorus ratio of 1.68∶1.

**Table 2:** Elemental analysis of ASP and Ca-ASP as determined by EDS spectroscopy.

Element	Ca Atomic%	P Atomic%	O Atomic%	C Atomic%	Ca/P Ratio of Atomic	C/O Ratio of Atomic
ASP	-	-	30.97	69.03	-	2.23
Ca-ASP	19.04	11.31	52.64	17.01	1.68	0.323

**Figure 4:**
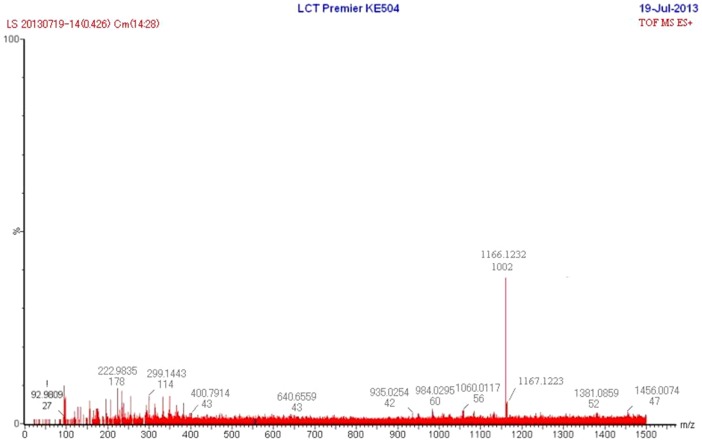
LC-MS scan pattern of Ca-ASP.

**Figure 5:**
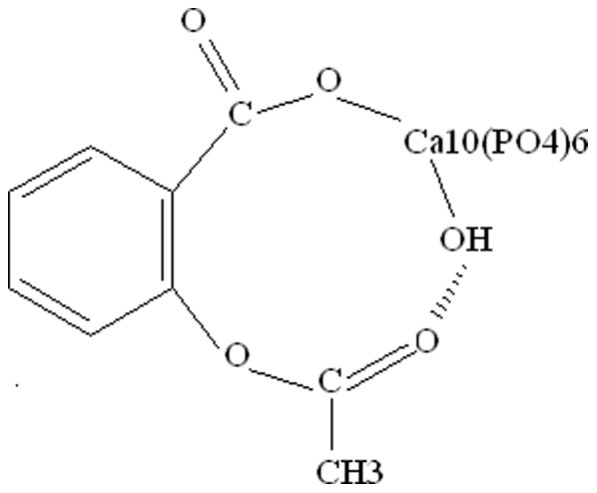
Schematic diagram of structure of Ca-ASP.

The molecular mass of Ca-ASP was measured by LC-MS, which gave a value of 1166.1645 (Figure 4). The molecular mass and the ratios of calcium to phosphorus and of calcium to oxygen corresponded to the structure of ASP-Hap, whereby one Hap molecule was conjugated to one ASP molecule, with the loss of a water molecule. As Ca-ASP was less soluble in water compared to ASP (3 g/L for ASP and 2.5 g/L for Ca-ASP), we predicted that it probably contains an ionic bond in addition to the hydrogen bond between the Hap and ASP components, consistent with the FTIR spectrum. Thus we proposed the structure of the Ca-ASP as shown in Figure 5.

### Antithrombus activity

#### Platelet count

**Figure 6:**
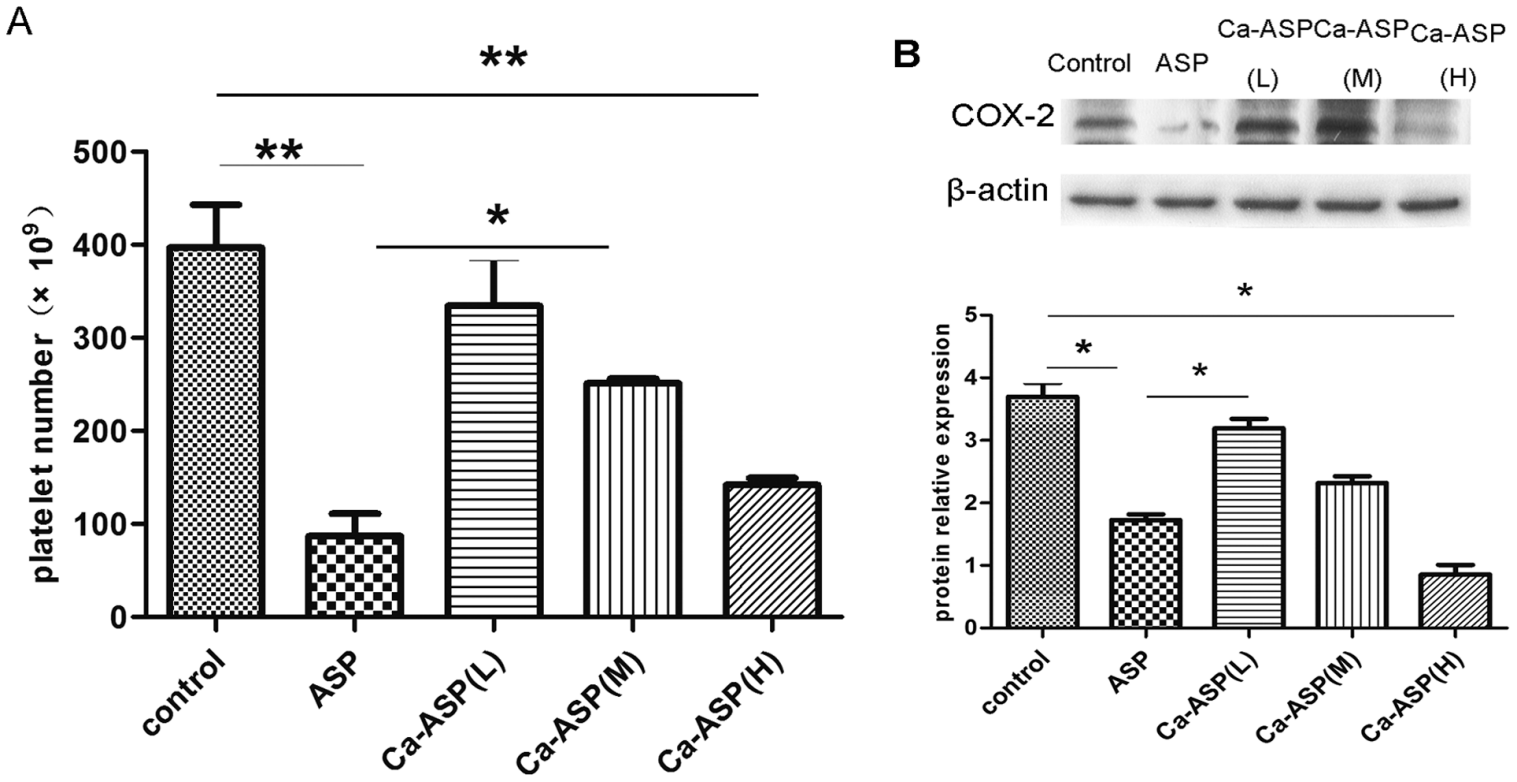
Analysis of platelet counts and COX-2 expression. (A) Platelet counts of rats underwent different treatments. (B) Western blot analysis of COX-2 expression in rats underwent different treatments.

Platelet count was used as an index for anti-thrombotic activity [26]. The result of platelet count is shown in Figure 6A. Rats administered ASP (pure aspirin) or Ca-ASP all had lower platelet counts than control rats. The highest level of anti-thrombotic activity was seen in the group given ASP, as shown by its lowest platelet count. At the other end of the spectrum, the control had the lowest level of anti-thrombotic activity, as seen from its highest platelet count. The difference in platelet count between these two groups was significant (*P*<0.01). The platelet counts of rats administered different doses of Ca-ASP were also lower than that of control rat. However, only those administered moderate (Ca-ASP/M) or high (Ca-ASP/H) dosage had significantly lower platelet counts than the control. Rats given low dosage of Ca-ASP showed no significant decrease in platelet count compared to the control. Among the three groups that received Ca-ASP, only the group receiving a high dose attained comparable platelet count with the group given ASP. These results showed that a moderate dose of Ca-ASP could increase the level of antithrombotic activity, but only by increasing the dose to that of ASP (0.5 mmol/kg) would the enhancement of antithrombotic activity achieved by Ca-ASP reach a similar level as that produced by ASP.

#### Expression of COX-2 in gastric tissue

Consistent with the result of platelet count, rats administered ASP or Ca-ASP had significantly lower levels of COX-2 compared to control rats, with the Ca-ASP(H) group showing the lowest level (Figure 6B). The level of COX-2 obtained for the Ca-ASP(H) group was about half the level of the ASP group, suggesting that Ca-ASP could better inhibit the expression of COX-2 than an identical dose of ASP. Therefore, a high dose of Ca-ASP could give a similar effect as ASP in terms of antithrombotic activity and inhibition of COX-2 expression. On the basis of these data, a high dose of Ca-ASP was chosen for subsequent experiments.

### Gastric mucosa ulcer index (UI)

**Figure 7:**
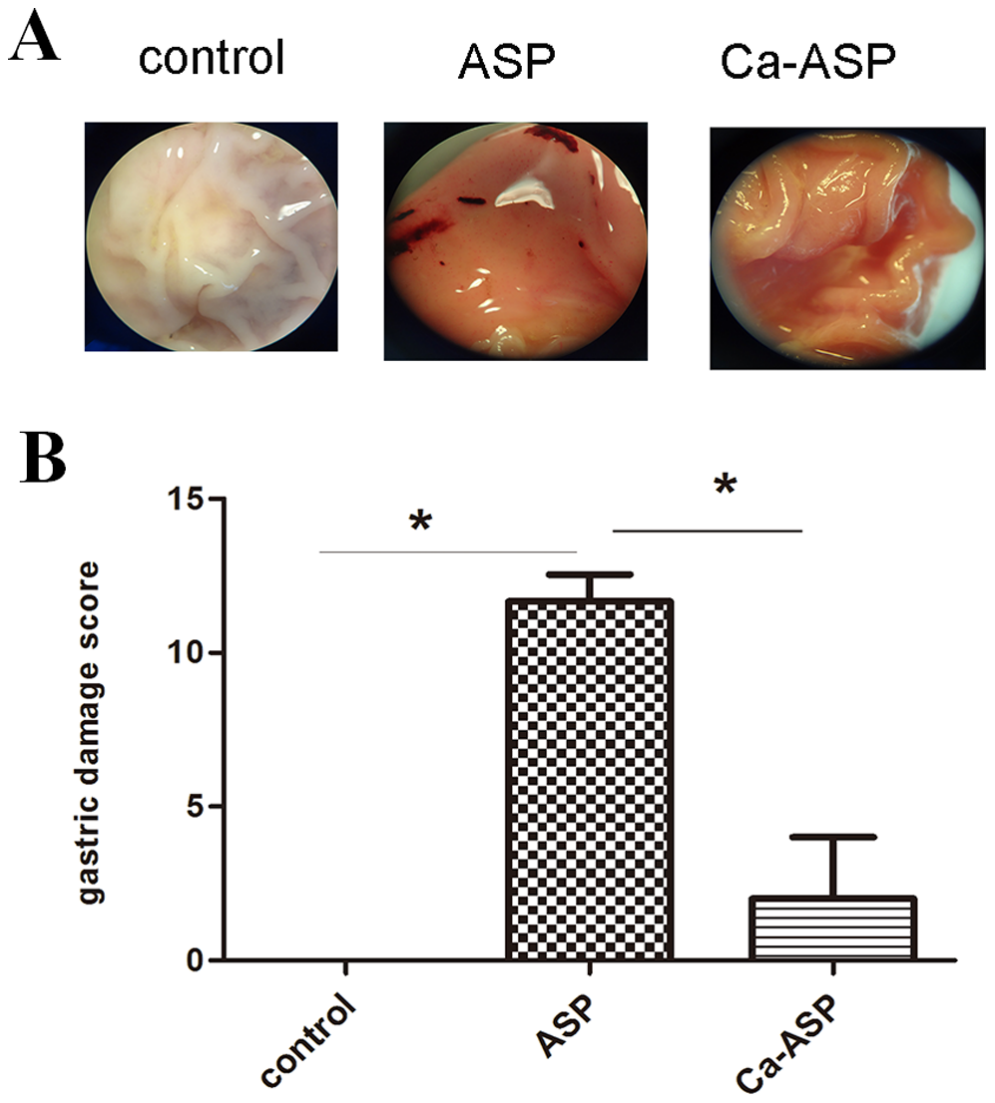
Effect of ASP and Ca-ASP on gastric mucosa of rats. (A) Photographs taken from microscopic examination of the gastric mucosa of mice underwent different treatments. (B) Comparison of the median ulcer index obtained for the gastric mucosa.

The extents of damage caused to the gastric mucosal surface by ASP and Ca-ASP were examined microscopically and assigned a score of median ulcer index. Compared to control rats, the mucosal surface of mice administered Ca-ASP exhibited slight damage, which was visible to the naked eyes, and was assigned a gastric damage score of 2 (Figure 7). Rats administered ASP on the other hand showed extensive sign of damage to the gastric mucosal surface, with patches of bleeding. The level of gastric mucosal damage in these animals was assigned a gastric damage score of 12.5. The reduction in gastric damage produced by Ca-ASP was significant, and this indicated that Ca-ASP had much less side effect than pure ASP.

### Mechanism of gastric mucosa protection of Ca-ASP

The mechanism associated with protection of gastric mucosa by Ca-ASP was investigated by determining the expression of COX-1 and PGE-2 in the gastric mucosal tissue.

#### Determination of the COX-1 level

**Figure 8:**
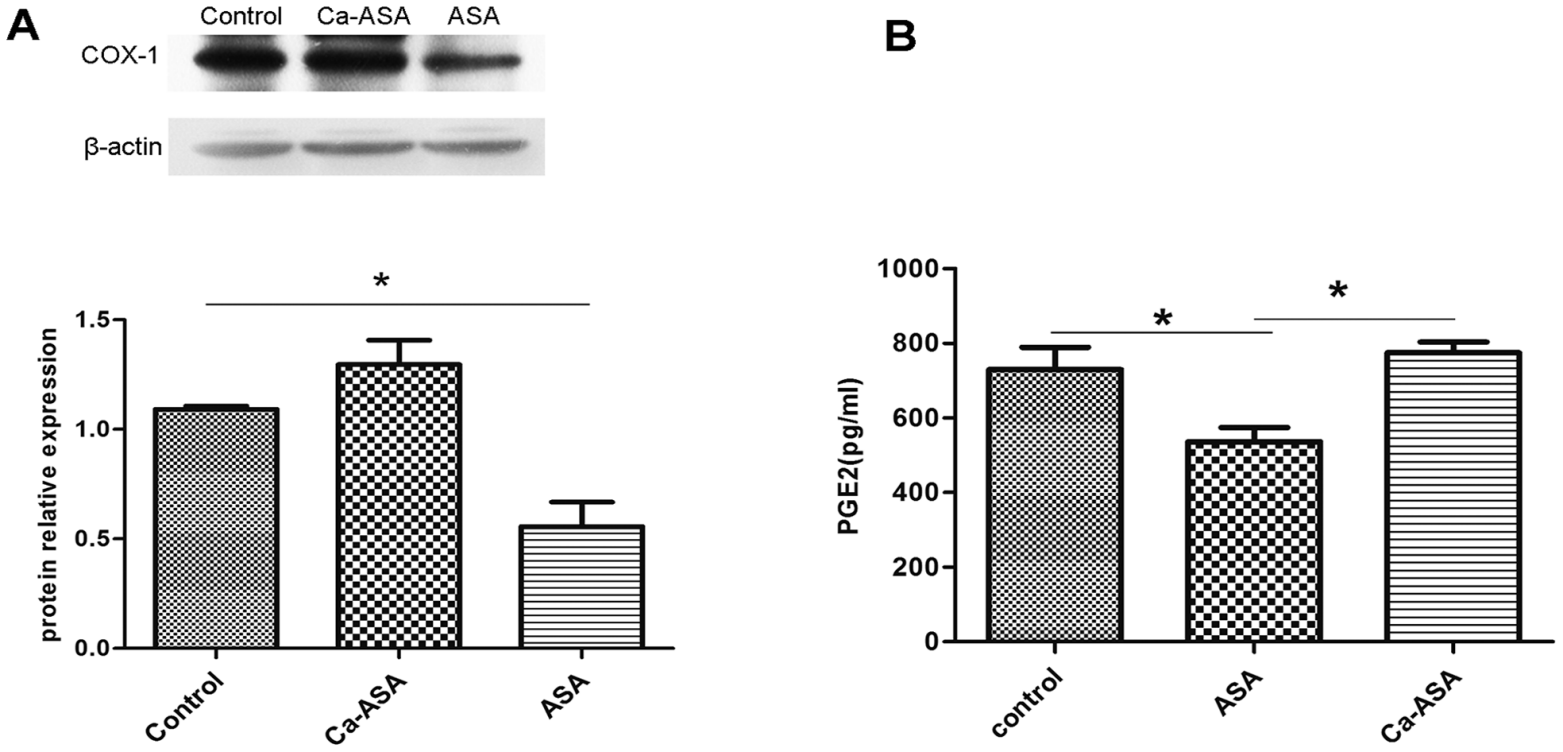
Effect of ASP and Ca-ASP on COX-1 expression (A) and PGE2 level (B) in rat gastric tissue.

The level of COX-1 expression in the gastric mucosa of rats administered a high dose of Ca-ASP was more than twice the level of rats given pure ASP (Figure 8A), and was also higher than the level of control rats, although this increase was not statistically significant. The results indicated that ASP inhibited while Ca-ASP not only did not inhibit the expression of COX-1 in the gastric mucosa. In contrast, Ca-ASP slightly improved the expression of COX-1 over that of control.

#### Determination of Gastric PGE2 level

Similarly, the level of gastric tissue PGE2 in rats administered Ca-ASP was similar to the level of control rats, but higher than that of rats given pure ASP, and the difference was significant (Figure 8B). Thus ASP appeared to inhibit the synthesis of PGE2 in the gastric tissue while an identical dose of Ca-ASP had no effect, and this was due to the lack of inhibitory effect exerted on the synthesis of COX-1 by Ca-ASP.

## Discussion

### Structure and composition of Ca-ASP

Since ASP has one carboxyl (COOH) group that is weakly acidic and Hap has two hydroxyl (OH) groups that are weakly basic, one would expect that the final product formed from the reaction of ASP with Hap to be (ASP)_2_-Hap, in which two ASP molecules are conjugated to one Hap molecule. However, the result of elemental analysis of Ca-ASP revealed a structure that consisted of one ASP conjugated to one Hap molecule in the form depicted in Figure 5. The reaction between ASP and Hap took place between the COOH group of ASP and the OH group of Hap via an acid-base type reaction. Although Hap has two OH groups, only one molecule of ASP was conjugated to it in Ca-ASP. This could be because a second molecule of ASP was prevented from reacting with the other OH group of Hap in Ca-ASP as a result of steric hindrance. Furthermore, the OH group of Hap formed an intramolecular hydrogen bond with the carbonyl (acetyl) group [C = O(CH_3_)] of ASP, which was probably more stable than if it were conjugated to another molecule of ASP.

### Antithrombus activity

The antithrombotic activity of ASP and Ca-ASP was supported by their inhibition of COX-2 expression in the gastric tissue (Figure 6B). COX-2 is the enzyme that synthesizes prostaglandin I2 (PGI2), and PGI2 is the antagonists of thromboxane (TXA2), which promotes the formation of cruor and buildup of plaques in the arteries that can further progress to thrombosis. Ca-ASP inhibited COX-2 expression to a comparable extent as ASP, when the dose given was the same for each compound (Ca-ASP/H in Figure 6B). This suggested that Ca-ASP had similar antithrombotic activity as ASP, and could therefore replace ASP if it also causes less damage to the gastric mucosa.

### Gastric mucosa protection of Ca-ASP

Comparing the gastric damage in rats inflicted by Ca-ASP to that inflicted by ASP, the damage inflicted on the gastric mucosa by Ca-ASP was much less than that inflicted by ASP, and this may be due to a number of possible factors. Firstly, the modification of ASP by Hap converted the free carboxyl (COOH) group of ASP to a salt form, making it less reactive, and the drug therefore caused no local lesion to the gastric mucosa. Secondly, Ca-ASP inhibited the expression of COX-2 but not COX-1, unlike ASP which inhibited the expression of both enzymes. Although COX-1 and COX-2 both synthesize prostaglandins using the substrate arachidonic acid, COX-1 maintains normal gastric mucosa. The conversion of arachidonic acid to PGH2 is catalyzed by both COX-1 and COX-2. PGH2 is a common substrate for further conversion into PGE2, PGI2, PGD2, PGF2 and TXA2 by a series of specific isomerase and synthase enzymes [27]–[28]. One of these enzymes, the cytoplasmic prostaglandin E synthase (cPGES) is able to convert COX-1-synthesized but not COX-2 synthesized PGH2 into PGE2. PGE2 is a recognized vasodilator factors *in vivo*, and it can inhibit platelet aggregation and thrombosis, accelerate the flow of the gastric mucosal microcirculation, promote the secretion of bicarbonate, mediate the adaptive immune protective function, increase protein synthesis and cell renewal, and finally enhance the repair ability of the damaged gastric mucosa [29]. Thirdly, Hap increased the synthesis of PGE2, which in turn could counteract its inhibition by ASP. Studies in recent years have shown that impaired microcirculation is one of the pathological reasons for the damage inflicted on the gastric mucosal barrier, which is accompanied by elevated levels of ET-1, and declining levels of NO and PGE2 in the blood and the gastric mucosa [30]. Thus the design of our ASP derivative exploited the function of Hap to stimulate PGE2 synthesis by conjugating ASP to Hap, so that following gastric absorption the Hap component may fulfill its function, the enhancement of PGE2 synthesis. Finally, endogenous Ca^2+^ in the gastric mucosa is an essential protective agent for the gastric mucosa and mucus, but it is easily coordinated with drug molecules that have strong coordination ability, such as ASP, and this would deplete the pool of Ca^2+^ in the gastrointestinal tract and deprive the gastric mucosa of the protection afforded by Ca^2+^ [31]. Thus by exposing ASP to a Ca^2+^-containing compound, Hap, ASP presumably became pre-coordinated with Ca^2+^, making it unable to coordinate with additional Ca^2+^ present in the Ca^2+^ pool in the gastrointestinal tract. However, we did not have any experimental evidence to show this phenomenon, and at this stage, would remain only as speculation. We hope to demonstrate this in our future study.

## Conclusions

In this study we modified ASP by reaction with Hap (a Ca^2+^-containing phosphate compound) to give a novel ASP derivative, Ca-ASP. The composition and structure of Ca-ASP were determined. Ca-ASP produced similar antithrombotic activity as ASP when administered to rats at the same dose as ASP (0.5 mmol/kg). However, Ca-ASP caused much less damage to the gastric mucosa by inhibiting COX-1 but not COX-2 expression, stimulating PGE2 synthesis (probably through the Hap component), and possibly by its lack effect on Ca^2+^ pool in the gastrointestinal tract. The absence of free carboxyl group in the ASP component in Ca-ASP may also contributed to its lack of side effect on the gastric mucosa. Future work will explore these mechanisms further to obtain a more comprehensive mechanism underlying the reduction of side effect associated with this new ASA derivative.
